# Inflammatory Markers, Pulmonary Function, and Clinical Symptoms in Acute COVID-19 Among Non-Hospitalized Adolescents and Young Adults

**DOI:** 10.3389/fimmu.2022.837288

**Published:** 2022-02-09

**Authors:** Lise Lund Berven, Joel Selvakumar, Lise Havdal, Tonje Stiansen-Sonerud, Gunnar Einvik, Truls Michael Leegaard, Trygve Tjade, Annika E. Michelsen, Tom Eirik Mollnes, Vegard Bruun Bratholm Wyller

**Affiliations:** ^1^ Department of Paediatrics, Akershus University Hospital, Lørenskog, Norway; ^2^ Institute of Clinical Medicine, University of Oslo, Oslo, Norway; ^3^ Department of Clinical Molecular Biology (EpiGen), Akershus University Hospital, Lørenskog, Norway; ^4^ Department of Pulmonary Medicine, Akershus University Hospital, Lørenskog, Norway; ^5^ Department of Microbiology and Infection Control, Akershus University Hospital, Lørenskog, Norway; ^6^ Fürst Medical Laboratory, Oslo, Norway; ^7^ Research Institute of Internal Medicine, Oslo University Hospital (Rikshospitalet), Oslo, Norway; ^8^ Department of Immunology, University of Oslo, Oslo, Norway; ^9^ Oslo University Hospital, Oslo, Norway; ^10^ Research Laboratory, Nordland Hospital, Bodø, Norway; ^11^ Centre of Molecular Inflammation Research, Norwegian University of Science and Technology, Trondheim, Norway

**Keywords:** SARS-CoV-2, cohort study, inflammatory markers, adolescents, non-hospitalized

## Abstract

**Summary:**

Mild, subacute COVID-19 in young people show inflammatory enhancement, but normal pulmonary function. Inflammatory markers are associated with age and male sex, whereas clinical symptoms are associated with age and female sex, but not with objective disease markers.

**Background:**

Coronavirus Disease 2019 (COVID-19) is widespread among adolescents and young adults across the globe. The present study aimed to compare inflammatory markers, pulmonary function and clinical symptoms across non-hospitalized, 12 – 25 years old COVID-19 cases and non-COVID-19 controls, and to investigate associations between inflammatory markers, clinical symptoms, pulmonary function and background variables in the COVID-19 group.

**Methods:**

The present paper presents baseline data from an ongoing longitudinal observational cohort study (Long-Term Effects of COVID-19 in Adolescents, LoTECA, ClinicalTrials ID: NCT04686734). A total of 31 plasma cytokines and complement activation products were assayed by multiplex and ELISA methodologies. Pulmonary function and clinical symptoms were investigated by spirometry and questionnaires, respectively.

**Results:**

A total of 405 COVID-19 cases and 111 non-COVID-19 controls were included. The COVID-19 group had significantly higher plasma levels of IL-1β, IL-4, IL-7, IL-8, IL-12, TNF, IP-10, eotaxin, GM-CSF, bFGF, complement TCC and C3bc, and significantly lower levels of IL-13 and MIP-1α, as compared to controls. Spirometry did not detect any significant differences across the groups. IL-4, IL-7, TNF and eotaxin were negatively associated with female sex; eotaxin and IL-4 were positively associated with age. Clinical symptoms were positively associated with female sex and age, but not with objective disease markers.

**Conclusions:**

Among non-hospitalized adolescents and young adults with COVID-19 there was significant alterations of plasma inflammatory markers in the subacute stage of the infection. Still, pulmonary function was normal. Clinical symptoms were independent of inflammatory and pulmonary function markers, but positively associated with age and female sex.

## Introduction

Severe acute respiratory syndrome coronavirus 2 (SARS-CoV-2) is a single-stranded RNA virus responsible for the coronavirus disease 2019 (COVID-19) pandemic, which has caused morbidity and mortality all over the world (1). The primary clinical manifestation of severe COVID-19 is pneumonia, which may progress into multi-organ failure and death (1).

The pathophysiology of COVID-19 is incompletely understood, but has been linked to a disrupted and disproportionate response of the immune system, particularly cytokine production (2, 3). Uncontrolled release of pro-inflammatory cytokines, such as Interleukin (IL)-1β (4), IL-6 (5, 6), IL-8 (4), IL-17 (7) and Tumour Necrosis Factor (TNF) (4, 8), by immune and non-immune effector cells is thought to contribute to the symptoms and severity of the disease (9). Additionally, the role of complement activation is increasingly recognized (10, 11). Data regarding the adaptive immune responses in COVID-19 are limited, but reduction and functional exhaustion of T cells during SARS-CoV-2 infection have been reported, and growing evidence suggests immunosuppressive abilities of SARS-CoV-2 of the adaptive immune responses (12–14).

Age is an important determinant of disease severity, and the majority of infected young individuals experience mild disease that does not require hospitalization (15, 16). Still, the possible life-threatening multisystem inflammatory syndrome in children (MIS-C) is recognized as a specific pediatric complication of SARS-CoV-2 infection (17). The pathophysiology of MIS-C is largely unknown, but is generally attributed to a “cytokine storm” analogous to observations in critically diseased adults. In addition, young individuals with mild disease seem to be equally at risk as hospitalized patients for developing persistent fatigue, dyspnoea, “brain fog” and other symptoms (often referred to as post-COVID syndrome) (18). In addition to age, sex is an important risk determinant; men are over-represented among patients with severe acute disease, presumably due to differences in the elicited immune responses (19), whereas women are at greater risk for developing post-COVID syndrome (20).

A general limitation of many previous studies is that they rely on hospitalized patients. Hence, knowledge of disease mechanisms in less severely affected individuals is disproportionally scarce. However, to understand differences in pathophysiological responses possibly accounting for the wide scatter of disease severity, which in turn may inform treatment and risk stratification, studies of young, non-hospitalized SARS-CoV-2 infected individuals are necessary.

This paper presents results from the baseline visit of an ongoing longitudinal cohort study on COVID-19 in non-hospitalized adolescents and young adults. To the best of our knowledge, this is by far the largest cohort from this specific population published to date. The aims of the present paper were: a) To compare inflammatory responses, pulmonary function tests and clinical symptoms across SARS-CoV-2 positive (cases) and SARS-CoV-2 negative (control) individuals. b) To explore associations between the inflammatory markers, clinical symptoms, pulmonary function and background variables in sub-acute SARS-CoV-2 infection.

## Materials and Methods

### Study Design

**Figure 1:**
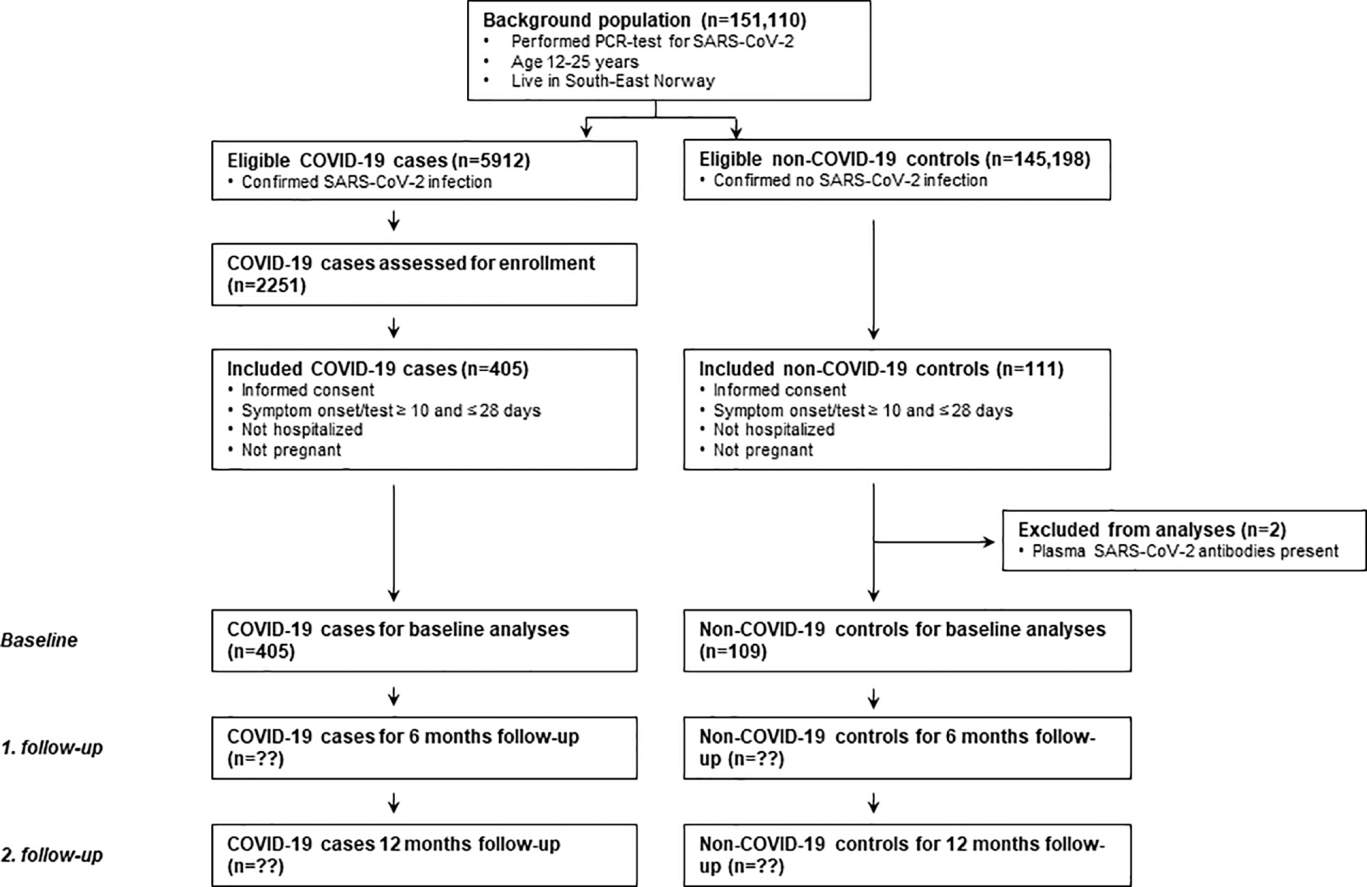
Flowchart of the LoTECA project (Long-Term Effects of COVID-19 in Adolescents). The present study report findings from baseline investigations. Follow-up is ongoing.

The Long-Term Effects of COVID-19 in Adolescents (LoTECA) project is a longitudinal observational cohort study of SARS-CoV-2 positive and negative non-hospitalised adolescents and young adults, with a total follow-up time of 12 months (**Figure 1**) (Clinical Trials ID: NCT04686734). In this paper, results from the baseline visit are reported. The project has been approved by the Norwegian National Committee for Ethics in Medical research.

### Participants

From December 24., 2020, until May 18., 2021, individuals 12-25 years old were consecutively recruited from two accredited microbiological laboratories (Fürst Medical Laboratories; Dept. of Microbiology and Infection Control, Akershus University Hospital), serving the counties of Oslo and Viken, Norway. During the first five weeks of the recruitment period, different genetic variants of the SARS-CoV-2 virus belonging to the B.1 lineage were present in this geographical area. From late-February 2021, the B.1.1.7 (alpha) variant became dominant for the remaining part of the recruitment period. Vaccination against COVID-19 was not routinely offered to the adolescents/young adult in this period.

Individuals with laboratory-confirmed SARS-CoV-2 infection (detected by upper respiratory tract swabs followed by reverse-transcription polymerase chain reaction (RT-PCR)) were eligible for enrolment after completed quarantine (10 days). Individuals having approximately the same distribution of sex and age as the SARS-CoV-2-infected cases, but with a negative SARS-CoV-2 test from the same microbiological laboratories during the same time period, were recruited as controls. Written informed consent was obtained from all participants or their legal guardians. Exclusion criteria were a) More than 28 days since onset of symptoms or SARS-CoV-2 test; b) Hospitalization due to COVID-19; c) Pregnancy (cf. **Supplementary Material** for details).

### Investigational Program

Participants were summoned to a one-day investigational program at our study centre at Akershus University Hospital, Norway (cf. **Supplementary Material** for details). Only a selection of variables is reported in the present paper.

### Blood Sampling and Laboratory Assays

Blood samples were obtained from antecubital venous puncture. EDTA whole blood samples were placed on ice-water for 5-60 minutes; thereafter, plasma was separated by centrifugation (2200 g, 10 min.) and frozen at –80°C until assayed.

Plasma samples were analysed using a multiplex cytokine assay (Bio-Plex Human Cytokine 27-Plex Panel; Bio-Rad Laboratories Inc., Hercules, CA, USA) containing the following cytokines: IL-1β, IL-1 receptor antagonist (IL1-ra), IL-2, IL-4, IL-5, IL-6, IL-7, IL-8, IL-9, IL-10, IL-12, IL-13, IL-15, IL-17A, eotaxin, basic fibroblast growth factor (bFGF), granulocyte-colony stimulating factor (G-CSF), granulocyte macrophage colony stimulating factor (GM-CSF), interferon (IFN)-γ, interferon-inducible protein (IP-10), monocyte chemotactic protein (MCP-1), macrophage inflammatory protein (MIP)-1α, MIP-1β, platelet derived growth factor-BB (PDGF-BB), regulated upon activation T cell expressed and secreted (RANTES), TNF, and vascular endothelial growth factor (VEGF). The samples were analysed on a Multiplex Analyser (Bio-Rad Laboratories) according to instructions from the manufacturer.

Plasma levels of growth/differentiation factor (GDF)-15 and C-reactive protein (CRP) were measured in duplicate by enzyme immunoassays (EIA) using commercially available antibodies (R&D Systems, Minneapolis, MN, USA) in a 384 format using a combination of a SELMA (Jena, Germany) pipetting robot and a BioTek (Winooski, VT) dispenser/washer. Absorption was read at 450 nm with wavelength correction set to 540 nm using an ELISA plate reader (Bio-Rad, Hercules, CA).

The complement activation products C3bc and the terminal complement complex (TCC) sC5b-9 were quantified in plasma using enzyme-linked immunosorbent assays (ELISAs) based on monoclonal antibodies designed against neoepitopes of the products, not reacting with the native component, and performed as described in detail previously (21). The units of these two well-established in-house assays are given according to an international standard defined as complement activation units (CAU) per millilitre with blood donors to define upper reference values of the normal population (21).

Serum samples were tested with the Elecsys^®^ Anti-SARS-CoV-2 immunoassay (Roche Diagnostics, Cobas e801, Mannheim, Germany) for IgG/IgM against the SARS-CoV-2 nucleocapsid antigen. The specificity and the sensitivity of the test are estimated by the manufacturer as 99.8% and 99.5%, respectively. Routine blood analyses of haematology and biochemistry were carried out the accredited laboratory at Akershus University Hospital.

### Spirometry

Spirometry was conducted to measure the forced vital capacity (FVC) and the forced expiratory volume in one second (FEV_1_) (EasyOne^®^ Air spirometer, EasyOne Connect software, NDD Medizintechnic AG, Switzerland). The ratio of FEV_1_/FVC was calculated. Procedures were executed according to the American Thoracic Society and European Respiratory Society guidelines, and recordings that did not adhere to technical quality requirements were excluded from the main analysis (22). The Global Lung Function Initiative 2012 network reference values were used to calculate the percentage of predicted values and the lower limit of normal (LLN) (23).

### Questionnaire

As to symptoms of acute SARS-CoV-2 infection, a previously developed inventory was slightly modified to fit the present patient group (24). The inventory consists of 24 symptoms that may be associated with COVID-19 (25), which the participants are asked to grade during the period from symptom onset/SARS-CoV-2 test until the present day. Grading is rated on five-point Likert scales from “never/rarely present” to “present all of the time”. The sum score across five items (fever/chills, sore throat, headaches, muscle ache and fatigue after exercise) was selected to represent general infectious symptoms (total range from 5 – 25), whereas the sum score across the items breathlessness, coughing and running nose was taken to represent airways symptoms (total range from 5 to 15).

### Statistical Analysis

A total number of approximately 400 COVID-19 cases and 100 non-COVID controls yields a power of 80% to detect small-to-medium effect sizes (Cohen’s *d*~0.30, α=0.05) in cross-sectional analyses. All statistical analyses were carried out in SPSS (SPSS Inc., Chicago, IL). Eight cytokines (IL-1ra, IL-5, IL-6, IL-10, IL-15, G-CSF, PDGF-BB and VEGF) had a large amount of missing data, and were excluded from further analyses. Cytokine values below lower detection limit (LDL) were replaced with a random value in the interval between zero and LDL for each specific cytokine. Plasma samples were missing from a total of 19 participants (16 COVID-19 cases, three non-COVID controls); these were not imputed, nor were missing data for other variables.

Variables are reported with mean/standard deviation or median/interquartile range and corresponding confidence intervals, depending on distribution. Cross-sectional comparisons were carried out by applying Student t, Mann-Whitney, χ^2^, or Fisher exact tests as appropriate. Associations between variables were explored by the non-parametric statistics Spearman’s rho.

A p-value < 0.05 was considered statistically significant (two-sided tests). As several variables were strongly correlated (e.g. the majority of the inflammatory markers), p-values were not adjusted for test multiplicity.

## Results

A total of 151,110 RT-PCR-tests of SARS-CoV-2 were carried out in individuals 12-25 years old at our two collaborating microbiological laboratories during the recruitment period of the present study (**Figure 1**). A total of 5912 (3.9%) of the tests were confirmative of SARS-CoV-2 infection; fraction of males was 51.0%. Of the confirmed cases, a total of 2251 individuals (50.5% males) were invited into the study.

A total of 405 SARS-CoV-2 positive cases and 111 SARS-CoV-2 negative controls fit the eligibility criteria and consented to participation. Within the control group, two individuals had detectable total antibody-titre (IgG/IgM) against SARS-CoV-2, and these were excluded from further analyses; thus the sample carried over to analyses consists of 405 COVID-19 cases (39.5% males, mean age 17.8 years) and 109 non-COVID controls (34.9% males, mean age 17.7 years) (**Table 1** **)**. Within the COVID-19 group, a median of 18 days passed between first symptom/positive SARS-CoV-2 test and inclusion.

**Table 1:** Background characteristics.

	*COVID-19 (n = 405)*	*Non-COVID (n = 109)*	p-value*
Sex - no. of males (%)	160 (39.5)	38 (34.9)	0.780
Age, years - median (range)	17.8 (12.9)	17.7 (12.3)	0.124
BMI, kg/m^2^ - mean (SD)	22.8 (4.4)	22.6 (4.2)	0.772
Days since symptom onset/postive test - median (range)	18 (22)	n.a.	n.a.
Serum SARS-CoV-2 total antibody titer** -.median (IQR)	6.7 (16.2)	n.a.	n.a.
Tympanic temperature, °C - mean (SD)	36.76 (0.38)	36.65 (0.36)	**0.008**
Respiratory frequency, breath/min - mean (SD)	16.7 (5.1)	16.7 (3.7)	0.967
SaO_2_, % - mean (SD)	98.6 (1.2)	98.6 (1.2)	0.575
Blood pH - mean (SD)	7.36 (0.03)	7.36 (0.03)	0.492
Blood *p*CO_2_, kPa - mean (SD)	6.3 (0.7)	6.3 (0.8)	0.353
Blood ${\text{HCO}}_{3}^{-}$ , mmol/L - mean (SD)	26.2 (1.8)	25.8 (1.9)	**0.036**
Blood Haemoglobin, g/dL - mean (SD)	13.5 (1.2)	13.6 (1.0)	0.491
Blood Platelet count, 10^9^ cells/L - mean (SD)	260 (57)	254 (50)	0.302
Blood Leukocyte count, 10^9^ cells/L - mean (SD)	5.9 (1.5)	5.6 (1.3)	**0.039**
Blood Lymphocyte count, 10^9^ cells/L - mean (SD)	2.1 (0.6)	2.1 (0.5)	0.402
Blood Monocyte count, 10^9^ cells/L - mean (SD)	0.46 (0.15)	0.42 (0.14)	**0.040**
Blood Neutrophil count, 10^9^ cells/L - mean (SD)	3.2 (1.2)	3.0 (1.0)	0.092
Neutrophil-to-Lymphocyte ratio – mean (SD)	1.6 (0.66)	1.5 (0.63)	0.405
Systemic immune-inflammation index (SII)*** - median (IQR)	373 (235)	365 (249)	0.361
Serum total IgG, g/L - mean (SD)	11.1 (2.2)	10.7 (2.0)	0.096
Serum total IgM, g/L - mean (SD)	1.3 (0.5)	1.2 (0.6)	0.111
Serum total IgA, g/L - mean (SD)	1.7 (0.8)	1.7 (0.7)	0.437

*Based upon Chi-square test, Mann-Whitney U test or Student t-test, as appropriate. **Total IgG/IgM against SARS-CoV-2 nucleocapsid antigen. ***SII=Neutrophils x Platelets/Lymphocytes. BMI, body mass index; n.a., not applicable; IQR, interquartile range; SD, standard deviation. P-values ≤0.05 are indicated with bold red.

### Comparison of COVID-19 Cases and Non-COVID Controls

The COVID-19 group had significantly higher plasma levels of IL-1β, IL-4, IL-7, IL-8, IL-12, TNF, IP-10, eotaxin, GM-CSF, bFGF, and the complement activation products TCC and C3bc, as compared to the non-COVID-19 group (**Table 2**). In particular, plasma levels of IL-1β and TCC were strikingly elevated, with a fold increase of 73 and 60, respectively. The plasma levels of IL-13 and MIP-1α were significantly lower in the COVID-19 group.

**Table 2:** Cytokines and complement activation markers.

	*COVID-19 (n = 389)*	*Non-COVID (n = 106)*	p-value*
Plasma hsCRP, µg/mL - median (IQR)	0.83 (2.2)	1.3 (3.0)	0.153
Confidence interval	0.73 to 1.1	0.72 to 1.7	
Plasma IL-1β, pg/mL - median (IQR)	0.63 (0.97)	0.0088 (0.22)	**<0.001**
Confidence interval	0.47 to 0.73	0.0072 to 0.19	
Plasma IL-2, pg/mL - median (IQR)	0.69 (1.9)	0.030 (1.6)	0.474
Confidence interval	0.47 to 1.1	0.030 to 0.78	
Plasma IL-4, pg/mL - median (IQR)	1.5 (0.80)	0.88 (0.74)	**<0.001**
Confidence interval	1.3 to 1.5	0.75 to 0.92	
Plasma IL-7, pg/mL - median (IQR)	12.6 (13.8)	4.8 (10.7)	**<0.001**
Confidence interval	11.5 to 12.6	2.1 to 5.7	
Plasma IL-8, pg/mL - median (IQR)	0.80 (2.0)	0.10 (0.18)	**<0.001**
Confidence interval	0.58 to 1.1	0.078 to 0.11	
Plasma IL-9, pg/mL - median (IQR)	68 (130)	70 (129)	0.595
Confidence interval	60 to 81	54 to 85	
Plasma IL-12, pg/mL - median (IQR)	1.5 (4.6)	0.19 (3.4)	**<0.001**
Confidence interval	1.4 to 1.5	0.15 to 1.1	
Plasma IL-13, pg/mL - median (IQR)	0.26 (0.56)	0.51 (1.1)	**<0.001**
Confidence interval	0.25 to 0.27	0.45 to 0.66	
Plasma IL-17A, pg/mL - median (IQR)	1.6 (2.7)	1.4 (2.4)	0.961
Confidence interval	1.3 to 2.0	0.69 to 2.0	
Plasma TNF, pg/mL - median (IQR)	7.5 (7.1)	4.3 (6.0)	**<0.001**
Confidence interval	6.7 to 8.2	3.0 to 5.4	
Plasma IFN-γ, pg/mL - median (IQR)	1.3 (1.6)	0.94 (1.2)	0.901
Confidence interval	1.0 to 1.3	0.94 to 1.1	
Plasma MCP-1, pg/mL - median (IQR)	12.3 (7.3)	12.5 (8.3)	0.116
Confidence interval	11.9 to 12.9	11.7 to 14.0	
Plasma IP-10, pg/mL - median (IQR)	149 (71)	115 (50)	**<0.001**
Confidence interval	141 to 156	106 to 123	
Plasma Eotaxin, pg/mL - median (IQR)	14.7 (7.4)	12.7 (6.3)	**0.001**
Confidence interval	14.0 to 15.2	11.6 to 13.6	
Plasma MIP-1α, pg/mL - median (IQR)	0.77 (0.40)	0.86 (0.39)	**0.001**
Confidence interval	0.67 to 0.82	0.79 to 1.0	
Plasma MIP-1β, pg/mL - median (IQR)	25 (38)	25 (34)	0.520
Confidence interval	22 to 27	21 to 28	
Plasma RANTES, pg/mL - median (IQR)	267 (404)	271 (347)	0.242
Confidence interval	237 to 295	230 to 310	
Plasma GM-CSF, pg/mL - median (IQR)	0.11 (0.58)	0.016 (0.019)	**<0.001**
Confidence interval	0.11 to 0.34	0.013 to 0.021	
Plasma bFGF, pg/mL - median (IQR)	3.4 (6.0)	1.3 (1.2)	**<0.001**
Confidence interval	2.7 to 3.4	1.1 to 1.5	
Plasma GDF-15, ng/mL - median (IQR)	0.37 (0.14)	0.36 (0.15)	0.354
Confidence interval	0.36 to 0.38	3.33 to 0.39	
Plasma TCC, CAU/mL - median (IQR)	0.18 (0.21)	0.0029 (0.17)	**<0.001**
Confidence interval	0.16 to 0.20	0.0023 to 0.060	
Plasma C3bc, ng/mL - median (IQR)	3.9 (2.3)	3.0 (1.3)	**<0.001**
Confidence interval	3.7 to 4.1	2.7 to 3.2	

*Based upon Mann-Whitney U-test. IQR, interquartile range; hsCRP, high-sensitive assay of C-reactive protein; IL, interleukin; TNF, tumor necrosis factor; IFN, interferon; MCP, Monocyte chemotactic protein; IP, Interferon gamma-induced protein; MIP, Macrophage inflammatory protein; RANTES, Regulated on activation, normal T-cell expressed and secreted; GM-CSF, granulocyte macrophage colony stimulating factor; bFGF, basic fibroblast growth factor; GDF, growth/differentiation factor; TCC, terminal complement complex; CAU, complement activation unit; C3b, complement component 3, part bc. P-values ≤0.05 are indicated with bold red.

Spirometry did not detect any significant differences in dynamic lung volumes between COVID-19 cases and non-COVID controls (**Table 3**). Similar results were found in a sensitivity analysis including all technically dubious recordings, except for a slightly increased fraction of individuals with FEV_1_ < LLN in the COVID-19 group (**Table 4**).

**Table 3:** Spirometry.

	*COVID-19 (n = 320)*	*Non-COVID (n = 98)*	p-value*
FVC, L - mean (SD)	4.2 (0.95)	4.2 (0.83)	0.704
Confidence interval	4.1 to 4.3	4.0 to 4.4	
FVC, % of predicted - mean (SD)	99.9 (9.9)	99.9 (10.5)	0.972
Confidence interval	98.8 to 101.0	97.8 to 102.0	
FVC < LLN - no. (%)	0 (0.0)	1 (1.0)	0.234
FEV1, L - mean (SD)	3.6 (0.74)	3.6 (0.67)	0.714
Confidence interval	3.5 to 3.7	3.5 to 3.7	
FEV1, % of predicted - mean (SD)	98.7 (10.1)	98.4 (9.9)	0.786
Confidence interval	97.6 to 99.8	96.4 to 100.4	
FEV1 < LLN - no. (%)	5 (1.6)	0 (0.0)	0.595
FEV1:FVC ratio - mean (SD)	0.86 (0.065)	0.86 (0.063)	0.929
Confidence interval	0.85 to 0.87	0.85 to 0.87	
FEV1:FVC ratio < 0.7 - no. (%)	7 (2.2)	2 (2.0)	1.000

*Based upon Student t-tests and Fisher’s excact test, as appropriate. SD=standard deviation; FVC, forced vital capacity; LLN, lower limit of normal; FEV1, forced expiratory volume 1 second. Individual spirometry recordings that did not safisify established quality criteria (22) were excluded from the analyses.

**Table 4:** Correlation (Spearman’s rho) between immunological markers, background variables, spirometry variables and clinical symptoms within the COVID-19 group (n = 389).

		*Days since test/symptom onset*	Sex*	*Age*	*FVC*	*FEV1*	*FEV1/FVC*	*General infectious symptoms*	*Airway symptoms*
Plasma IL-1β	*Corr. coeff. (rho)*	**-0.24**	-0.05	0.02	-0.02	-0.03	-0.01	-0.03	-0.01
	*p-value*	**<0.001**	0.320	0.760	0.694	0.573	0.823	0.564	0.786
Plasma IL-4	*Corr. coeff. (rho)*	-0.04	**-0.17**	**0.12**	**0.17**	**0.17**	0.00	0.07	0.09
	*p-value*	0.459	**0.001**	**0.017**	**0.001**	**0.001**	0.931	0.172	0.083
Plasma IL-7	*Corr. coeff. (rho)*	-0.07	**-0.11**	-0.01	0.08	0.10	0.04	-0.02	-0.03
	*p-value*	0.159	**0.028**	0.793	0.115	0.050	0.382	0.748	0.602
Plasma IL-8	*Corr. coeff. (rho)*	**-0.24**	-0.04	0.01	0.05	0.05	0.01	-0.02	-0.07
	*p-value*	**<0.001**	0.380	0.859	0.284	0.290	0.845	0.656	0.207
Plasma IL-12	*Corr. coeff. (rho)*	**0.15**	-0.08	0.00	0.02	0.03	0.00	-0.08	-0.06
	*p-value*	**0.003**	0.131	0.992	0.711	0.521	0.959	0.123	0.237
Plasma IL-13	*Corr. coeff. (rho)*	**-0.19**	-0.07	0.03	0.03	0.02	-0.05	-0.04	-0.02
	*p-value*	**<0.001**	0.180	0.566	0.556	0.658	0.329	0.489	0.710
Plasma TNF-α	*Corr. coeff. (rho)*	**-0.32**	**-0.16**	-0.06	0.06	0.02	-0.07	-0.04	-0.01
	*p-value*	**<0.001**	**0.002**	0.202	0.245	0.751	0.170	0.389	0.863
Plasma IP-10	*Corr. coeff. (rho)*	0.02	-0.03	0.09	0.02	0.00	-0.07	-0.02	0.03
	*p-value*	0.704	0.593	0.086	0.730	0.991	0.193	0.693	0.603
Plasma Eotaxin	*Corr. coeff. (rho)*	0.04	**-0.15**	**0.17**	**0.18**	**0.19**	0.02	0.01	0.00
	*p-value*	0.435	**0.003**	**0.001**	**0.001**	**<0.001**	0.639	0.875	0.955
Plasma MIP-1α	*Corr. coeff. (rho)*	-0.04	-0.02	0.04	0.05	0.03	-0.04	-0.01	-0.01
	*p-value*	0.488	0.711	0.466	0.310	0.523	0.458	0.816	0.836
Plasma GM-CSF	*Corr. coeff. (rho)*	**0.15**	-0.08	-0.02	**0.10**	**0.11**	-0.05	-0.05	0.01
	*p-value*	**0.004**	0.114	0.749	**0.042**	**0.038**	0.286	0.298	0.906
Plasma bFGF	*Corr. coeff. (rho)*	**0.20**	-0.03	0.03	0.03	0.04	0.05	0.03	-0.01
	*p-value*	**<0.001**	0.502	0.599	0.523	0.438	0.361	0.512	0.820
Plasma TCC	*Corr. coeff. (rho)*	-0.10	0.02	0.09	0.02	0.00	-0.06	0.02	-0.02
	*p-value*	0.050	0.661	0.077	0.694	0.961	0.204	0.730	0.675
Plasma C3bc	*Corr. coeff. (rho)*	0.07	0.03	0.00	-0.01	-0.03	-0.08	0.04	0.01
	*p-value*	0.147	0.585	0.996	0.840	0.561	0.140	0.473	0.878

*Male sex is reference category. IL, interleukin; TNF, tumour necrosis factor; IP, Interferon gamma-induced protein; MIP, Macrophage inflammatory protein; GM-CSF, granulocyte macrophage colony stimulating factor; bFGF, basic fibroblast growth factor; TCC, terminal complement complex; C3bc, complement component 3, part bc. FVC, forced vital capacity; FEV1, forced expiratory volume 1 second. P-values ≤0.05 and their related correlation coefficients are indicated with bold red.

All clinical symptoms were rated significantly higher among COVID-19 cases as compared to non-COVID-19 controls, except for sore throat and running nose (**Table 5**).

**Table 5:** Clinical symptoms.

	*COVID-19 (n = 390)*	*Non-COVID (n = 108)*	p-value*
Sensation of fever - mean (SD)	1.7 (1.0)	1.4 (0.7)	**0.004**
Confidence interval	1.6 to 1.8	1.2 to 1.5	
Sore throat - mean (SD)	1.9 (1.1)	1.6 (0.8)	0.063
Confidence interval	1.8 to 2.0	1.5 to 1.8	
Headache - mean (SD)	2.5 (1.3)	2.2 (1.1)	**0.047**
Confidence interval	2.4 to 2.6	2.0 to 2.4	
Muscle ache - mean (SD)	2.0 (1.3)	1.7 (1.0)	**0.015**
Confidence interval	1.9 to 2.1	1.5 to 1.9	
Unusual fatigue after physical activities - mean (SD)	2.5 (1.4)	1.7 (0.9)	**<0.001**
Confidence interval	2.3 to 2.6	1.5 to 1.8	
General infectious symptoms, total score - mean (SD)	10.5 (4.4)	8.5 (2.9)	**<0.001**
Confidence interval	10.1 to 11.0	8.0 to 9.1	
Breathlessness - mean (SD)	2.1 (1.3)	1.5 (0.7)	**<0.001**
Confidence interval	1.9 to 2.2	1.3 to 1.6	
Coughing - mean (SD)	2.5 (1.3)	1.8 (0.9)	**<0.001**
Confidence interval	2.4 to 2.6	1.6 to 2.0	
Running nose - mean (SD)	2.6 (1.3)	2.5 (1.2)	0.349
Confidence interval	2.5 to 2.7	2.2 to 2.7	
Airway symptoms, total score - mean (SD)	7.2 (2.9)	5.8 (2.0)	**<0.001**
Confidence interval	6.9 to 7.5	5.4 to 6.1	

*Based upon Mann-Whitney U-tests. General infectious symptoms total score has a range from 5 to 25. Airway symptoms total score has a range from 5 to 15. SD, standard deviation. P-values ≤0.05 are indicated with bold red.

Neither the systemic immune-inflammation index (SII) nor the neutrophil-to-lymphocyte ratio (NLR) revealed any significant differences between COVID-19 cases and non-COVID-19 controls (**Table 1**), thus, these markers were not subjected to further analyses. *Associations within the COVID-19 group*.

Within the COVID-19 group, IL-1β, IL-8, IL-13, and TNF were negatively associated whereas IL-12, GM-CSF and bFGF were positively associated with days since symptom onset/positive test (**Table 4**). Also, four inflammatory markers (IL-4, IL-7, TNF, eotaxin) were negatively associated with female sex; two of them (eotaxin, IL-4) were positively associated with age. No inflammatory markers were negatively associated with pulmonary function variables or positively associated with clinical symptoms.

Only one clinical symptom (muscle ache) showed a significant negative association with days since symptom onset/positive test (**Table 6**), whereas a majority of symptoms were positively associated with female sex and age. There were no associations between clinical symptoms and pulmonary function variables.

**Table 6:** Correlation (Spearman’s rho) between clinical symptoms, background variables, and spirometry variables within the COVID-19 group (n = 390).

		*Days since test/symptom onset*	Sex*	*Age*	*FVC*	*FEV1*	FEV1/FVC
Sensation of fever	*Corr. coeff. (rho)*	-0.07	**0.10**	0.07	-0.05	-0.04	0.02
	*p-value*	0.153	**0.045**	0.161	0.323	0.402	0.727
Headache	*Corr. coeff. (rho)*	-0.08	**0.31**	**0.25**	-0.06	-0.04	0.05
	*p-value*	0.128	**<0.001**	**<0.001**	0.238	0.404	0.300
Muscle ache	*Corr. coeff. (rho)*	**-0.13**	**0.26**	**0.21**	-0.06	-0.06	0.04
	*p-value*	**0.014**	**<0.001**	**<0.001**	0.268	0.234	0.438
Unusual fatigue after physical activities	*Corr. coeff. (rho)*	0.00	**0.25**	**0.27**	0.03	0.02	0.00
	*p-value*	0.982	**<0.001**	**<0.001**	0.570	0.692	0.925
General infectious symptoms, total score	*Corr. coeff. (rho)*	-0.07	**0.32**	**0.24**	-0.05	-0.05	0.06
	*p-value*	0.190	**<0.001**	**<0.001**	0.291	0.361	0.242
Dyspnoea	*Corr. coeff. (rho)*	0.06	**0.21**	**0.15**	-0.03	-0.04	0.03
	*p-value*	0.211	**<0.001**	**0.003**	0.541	0.419	0.557
Coughing	*Corr. coeff. (rho)*	-0.01	0.00	0.04	0.04	0.01	-0.10
	*p-value*	0.859	0.961	0.399	0.428	0.910	0.056
Airway symptoms, total score	*Corr. coeff. (rho)*	0.01	**0.15**	**0.13**	-0.01	-0.04	-0.05
	*p-value*	0.830	**0.003**	**0.010**	0.812	0.405	0.291

*Male sex is reference category. FVC, forced vital capacity; FEV1, forced expiratory volume 1 second. P-values ≤0.05 and their related correlation coefficients are indicated with bold red.

## Discussion

The present study of a large group of young, non-hospitalised COVID-19 patients show that: a) there are significant alterations of plasma inflammatory markers in the subacute stage of the infection, signalizing a relative persistence of the innate immune responses; b) some plasma inflammatory markers are positively associated with age and male sex; c) despite ongoing inflammatory activity, pulmonary function is not affected; d) clinical symptoms are largely independent of inflammatory and pulmonary function, but positively associated with age and female sex.

The findings of inflammatory marker elevation in plasma corroborate results from other studies reporting a strong increment of pro-inflammatory cytokines in adults as well as in the few paediatric studies that exists to date (17, 26–29). However, a striking feature of the present study is the lack of differences regarding cytokines that have been implicated as markers of disease severity, most notably IL-6 and IL-10 (30, 31). In an Italian cohort of 77 adult patients, IL-6 level at hospital admission was shown to be the best prognostic marker for negative outcomes in COVID-19 (30). Furthermore, Sun et al. reported an increased expression of IL-6, IL-10 and INF-γ in paediatric patients with severe COVID-19 (32). Also, a recent meta-analysis involving nine studies showed that mean serum level of IL-6 was more than three-fold higher in complicated COVID-19 cases, and was also associated with in-hospital mortality risk (31). In the present study, for both these cytokines, a majority of values in both groups were below the lower limit of detection (LLD), and they were therefore not subjected to formal statistical comparisons. Also, MCP-1, a predictor of disease severity in some hospital-based cohorts (17, 27), was not elevated in the present study. A plausible explanation for the lack of differences regarding these three markers is that we studied a non-hospitalised cohort. This resonates with data from seven paediatric studies evaluated by Soraya and Ulhaq (33). In all seven studies, the COVID-19 patients had relatively mild symptoms, and the IL-6 level tended to be within the normal range. Also, a meta-analysis by Zhang et al. revealed significantly higher levels of the cytokines TNF, IL-5, IL-6 and IL-10 and the chemokines MCP-1, IP-10 and eotaxin in severe cases in comparison to mild cases of COVID-19 (27). From a clinical point of view, it should be noted that, in the present paper, CRP levels were within normal range among COVID-19 patients as well, again as opposed to studies of patients with severe disease (28). It is therefore possible that CRP measurement, which belongs to the standard armamentarium of general practitioners, may be valuable in identifying patients at risk for a more severe course of the infection. The observation of a negative association between pro-inflammatory markers and days since symptom onset/SARS-CoV-2 testing combined with a positive association to IL-12 suggest that the inflammatory response subsides and is replaced by an adaptive immunological response during the first few weeks after the infectious event. Interestingly, recent evidence suggests that temporal difference in resolution of the innate immunological response is the main reason for a less severe disease course among children than adults (26). This also fits an observation in the present study of a positive association between the pro-inflammatory marker eotaxin and age.

In hospital-based cohorts, several previous studies suggest that complement activation, in particularly reflected by increased TCC, is a negative prognostic marker in acute COVID-19 among adults as well as children (34, 35). A recent prospective cohort study of 102 hospitalised and 26 non-hospitalised COVID-19 patients showed that increased complement activation was characteristic for hospitalised patients, and that complement activation was significantly associated with markers of inflammation, such as CRP and IL-6 (35). Interestingly, complement activation was a distinct feature of the present study as well, and did not seem to resolve over time. Complement factors have in general short half-life (seconds to a few hours) *in vivo*; thus, the present results suggest a continuous stimulus for complement activation, which should be scrutinized in future research project. The increase in TCC was among those with highest fold change, consistent with the fact that TCC has a substantially longer half-life than for instance C5a which is difficult to detect in increased amounts due to the very short half-life, but highly inflammatory potent (36). At the same time, the present results show that complement activation, detected by TCC, is not limited to severe cases of COVID-19. The reason for this might be that we have studied a younger population, and young people may be more resistant to increased complement activation than older individuals. Indeed, a study of 120 healthy Norwegians, 20-69 years old, demonstrated that there were significant age- and sex-related differences in complement levels and functionality (37). A particularly interesting observation regarding complement in this study was that the marker with the second largest fold increase after TCC was IL-1β, which is an important part of the NLRP3 inflammasome, a crucial actor in the inflammatory network (38, 39). Thus, these two very highly increased mediators link the complement and the inflammatory system by cross-talk that previously has been reviewed for complement and Toll-like receptors (40).

The present study indicated a positive association between three pro-inflammatory markers (IL-7, TNF, and eotaxin) and male sex. This may support differences in inflammatory responses as a main reason for a more severe course of acute disease among men than women (27), and should be further investigated in future research.

In some follow-up studies of hospital-based adult COVID-19 cohorts, persistent alterations of dynamic pulmonary function have been reported (41–44). Results are conflicting though, as a relatively large adult study did not find any substantial alterations of spirometry tests results despite persistent radiological abnormalities (45), and two small cases series reported spirometry to be normal or near-normal in the aftermath of COVID-19 in children (46, 47). The present study confirms that pulmonary function appears to be normal in non-severe cases of COVID-19 in adolescents and young adults, and there were no associations to inflammatory markers nor clinical symptoms. Thus, it seems unlikely that persistent respiratory symptoms (such as dyspnoea), reported to be common and linger for a long time after mild COVID-19 (20), are caused by deterioration of pulmonary function. We note that for a subset of 63 participants, technical criteria were not met, major reasons were that they were unable to exhale long enough, either due to cough or fatigue. This may theoretically be due to post-infective bronchial reactivity, which should be attended to in future research.

As expected, the present study confirmed a higher incidence of typical clinical symptoms among the COVID-19 cases. Surprisingly, these symptoms were not associated to inflammatory markers or spirometry variables, nor did they tend to subside over time. On the other hand, a majority of clinical symptoms correlated strongly with female sex and age. These observations seem to corroborate results from studies on post-COVID syndrome, where female sex is consistently reported to be a risk factor, but with scarce findings of inflammatory abnormalities (20, 48, 49). The apparent disconnection between clinical symptoms and biological aberrations is an intriguing observation that gives further merit to studies suggesting mental processes as the main determinant of symptom persistence after COVID-19 (50), and deserves further investigations.

Strengths of the present study include a large and well-defined group of non-hospitalised young individuals with COVID-19 and a comparable control group. Weaknesses include a somewhat skewed sampling of cases towards more females as compared to the background population. Also, for clinical symptoms, we did not ask the participants to grade the present state but rather the frequency over a defined time period, which may potentially explain the poor correlation between symptoms and disease markers.

In conclusion, non-hospitalised adolescents and young adults with acute COVID-19 showed activation of inflammatory markers during the subacute phase of the infection, of which some are positively associated with older age and male sex. Pulmonary function were normal, whereas clinical symptoms were independent of both inflammatory and pulmonary markers but associated with older age and female sex.

## Data Availability Statement

The raw data supporting the conclusions of this article will be made available by the authors, without undue reservation.

## Ethics Statement

The studies involving human participants were reviewed and approved by Regionale Komiteer for Medisinsk og Helsefaglig Forskningsetikk (REK). Written informed consent to participate in this study was provided by the participants’ legal guardian/next of kin.

## Author Contributions

The authors confirm contribution to the paper as follows: Study conception and design: VW. Data collection: LB, JS, LH, TS-S. Analysis and interpretation of results: LB, VW, JS, LH, TM, TT, AM, TM, GE. Draft manuscript preparation: LB, VW. All authors reviewed the results and approved the final version of the manuscript.

## Funding

This work was supported by the Norwegian Research Council [302079].

## Conflict of Interest

The authors declare that the research was conducted in the absence of any commercial or financial relationships that could be construed as a potential conflict of interest.

## Publisher’s Note

All claims expressed in this article are solely those of the authors and do not necessarily represent those of their affiliated organizations, or those of the publisher, the editors and the reviewers. Any product that may be evaluated in this article, or claim that may be made by its manufacturer, is not guaranteed or endorsed by the publisher.
