# Supplementary material for: Inflammatory Markers, Pulmonary Function, and Clinical Symptoms in Acute COVID-19 Among Non-Hospitalized Adolescents and Young Adults
Source: Front Immunol. 2022 Feb 9;13:837288. doi: 10.3389/fimmu.2022.837288 (PMC8864121; doi:10.3389/fimmu.2022.837288)
Supplement: Supplementary file 1 [file DataSheet_1.docx]

**Supplementary Material**

*Lund-Berven L, et al. Inflammatory markers, pulmonary function and clinical symptoms in acute COVID-19 among non-hospitalised adolescents and young adults*

**Study methods – supplementary details**

**Results – supplementary details**

*Suppl.Table 1. Spirometry with less rigorous exclusion criteria*

**Study methods – supplementary details**

**
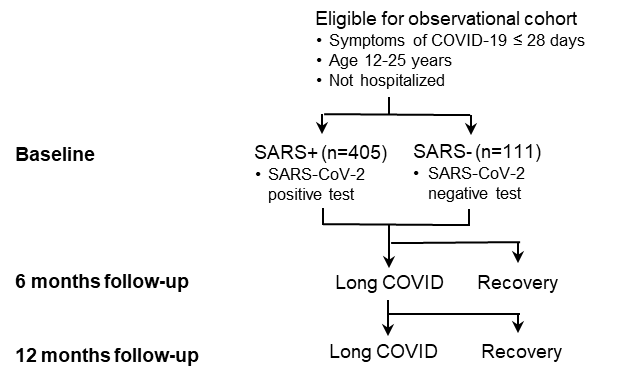
Overview of the LoTECA project**

Long-term Effects of Covid-19 in Adolescents (LoTECA) is a longitudinal observational cohort study of non-hospitalized adolescents and young adults (12-25 years) with confirmed SARS-CoV-2-infection (COVID+) (n=405) as well as refuted SARS-CoV-2-infection (COVID−) (n=111) (Fig. 3). The inclusion period lasted from Jan. 6 until May 28, 2021, and follow-up appointments are scheduled 6 and 12 months after inclusion. Hence, the entire data gathering will be completed by Q2/2022. At all time points, participants are subjected to a standardised investigational program (cf. details below):

- *Clinical assessment*: A comprehensive recording of exposures, previous medical history, symptoms and findings, and routine blood tests.

***Figure 1****. Simplified overview of the LoTECA design*

- *Biobanking*: Biobanking of blood (EDTA whole blood for DNA analyses, PAXgene-blood for mRNA-analyses, plasma, serum, viable peripheral blood mononuclear cells (PBMC)), urine, hair and fecal samples, for subsequent analyses.
- *Questionnaire*: Charting of clinical symptoms (e.g. fatigue, post-exertional malaise, sleep disturbances, pain, “brain fog”), as well as emotional disturbances (depression/anxiety, negative affect,), cognitive factors (worrying tendencies, self-efficacy), attentional factors (illness perception, interoception), personality traits (neuroticism, emotional awareness), and social factors (loneliness, adverse life events).
- *Functional testing:* Neuropsychological functions (learning, recall and recognition, working memory, attentional bias), cardiac function (ECG, heart rate variability, echocardiography), and pulmonary function (spirometry).

In addition to this standardized program, subgroups of individuals were recruited into three substudies featuring echocardiography, functional brain magnetic tomography (fMRI) and qualitative interview, respectively, as outlined in detail below.

**Recruitment, inclusion and exclusion**

Adolescents suffering from SARS-CoV-2 infection were recruited in close collaboration with Fürst Medical Laboratory and the Department of Microbiology and Infection Control at Akershus University Hospital. Laboratory staff reported regularly (three times weekly) to the LoTECA study center all confirmed SARS-CoV-2 infections (based upon RT-PCR of nasopharyngeal swab) in individuals between 12 and 25 years old living in the counties Oslo and Viken. Eligible individuals received a cell phone text message for information about the LoTECA project. Those who were interested in participating contacted a research coordinator at our study center who provided further information on the project, assessed inclusion and exclusion criteria (Table 1), and made a specific appointment for baseline investigations of those who were still interested and fulfilled all criteria. For practical reasons, the appointment was scheduled after the required isolation period (i.e. ≥10 days after first symptom/positive test result). All participants gave written informed consent; for those between 12 and 16 years, written informed consent was obtain from the parents/next-of-kin as well, according to Norwegian precepts.

Healthy adolescents having approximately the same distribution of sex and age as the SARS-CoV-2-infected individuals, but with a negative SARS-CoV-2 test during the same time period, were recruited using the same approach as with the SARS-CoV-2 positive cases (cf. above).

| **Table 1. Criteria for inclusion and exclusion** |  |
| --- | --- |
| **Inclusion criteria – cases** | **Inclusion criteria – controls** |
| Positive SARS-CoV-2 test | Suspected SARS-CoV-2 infection |
| Age 12-25 years | Negative SARS-CoV-2 test |
| ≤ 28 days since onset of first symptom | Age 12-25 years |
|  | ≤ 28 days since onset of first symptom |
|  |  |
| **Exclusion criteria – cases** | **Exclusion criteria – controls** |
| Hospitalised because of COVID-19 | Pregnancy |
| Pregnancy | Lack of written consent from patient/next-of-kin |
| Lack of written consent from patient/next-of-kin |  |

**Investigational program**

Participants were summoned to a one-day investigational program at Akershus University Hospital, Norway. All participants were instructed to abstain from tobacco products and caffeine at least 48 hours. Participants brought morning spot urine in a sterile container as well as fecal samples. Participants met in the morning or at noon. The entire program lasted for approximately two and a half hours.

**Clinical examination**

The clinical examination included a standardized review of organ systems, as well as biometrical measurements (body proportions, blood pressures, heart rate, respiratory rate, tympanic temperature, oxygen saturation). Routine blood samples for hematology and biochemistry were assayed at the accredited laboratory at Akershus University Hospital, Norway.

**Biobanking**

Biosamples included whole blood, plasma, serum, peripheral blood mononuclear cells (PBMC), feces, morning spot urine and hair. Samples that were not assayed immediately were frozen to -80⁰C within two hours. PBMC were stores at -150⁰C. Blood sampling was obtained in a fixed sequence from antecubital venous puncture. Blood samples for analyses of plasma biomarkers were collected in 4 mL EDTA tubes. They were immediately put on ice and centrifuged (2200 *g*, 10 minutes) within 15 minutes. Plasma was then transferred into cryotubes and frozen at -80 ⁰C. Blood samples for analysis of serum biomarkers were obtained in vacutainer tubes with gel from Sigma-Aldrich (St. Louis, MO, USA) and centrifuged (2200 *g*, 10 minutes) 30 minutes after collection. Serum was then transferred into cryotubes and frozen at -80 ⁰C.

Participants were instructed to bring a morning spot urine sample in a sterile container. The urine sample was assayed whit a Multistixs 5 (Siemens Healthcare, Erlangen, Germany), and for the female participants hCG levels was measured with InstAlert (Innovacon, San Diego, CA, USA), prior to further analyses.

Participants were instructed to bring fecal samples from the last three days using a fecal sample collection kit that ensures easy collection of fecal material on filter paper (Bio-Me, Oslo, Norway). The fecal samples were stored at -80⁰C.

A hair sample was collected from the parietal/occipital region of the scull: A bundle of hair with approximately the same diameter as a pencil was cut as close to the scalp as possible and frozen at -80⁰C.

**Questionnaires**

*Background*

Background variables included ethnicity, previous medical history, household members, and parents’ education; the latter was categorized according to the International Standard Classification of Occupations [1]. Furthermore, participants were asked to grade their consumption of alcoholic beverages, tobacco products and narcotics/illegal drugs on 5-point Likert scales, ranging from “never” to “every day/almost every day”.

*Present symptoms and functional abilities*

The Chalder Fatigue Questionnaire (CFQ) charts subjective experience of physical and mental fatigue, and has been extensively used in CFS research [2-4]. The CFQ has been translated and validated for a Norwegian population [5]. Total range is from zero to 33; higher scores imply more severe fatigue. Furthermore, fatigue caseness was defined as a CFQ total dichotomous score of 4 or higher (each item scored 0-0-1-1) [6].

Post-Exertional Malaise (PEM) is charted using five items from the validated DePaul Symptom Questionnaire. Total score is from 0 to 100 [7].

The Brief Pain Inventory (BPI) is a validated questionnaire for assessing pain [8]. In this study, the four BPI items assessing pain severity on ten-point Likert scales were used to compute a total sum score; in addition, scores on a single item (average pain) are reported.

The Karolinska Sleep Questionnaire (KSQ) is a validated questionnaire charting insomnia and other sleep disturbances. It consists of 14 items scored one to six on Likert scales [9]. Indexes for insomnia, awakening problems, and sleepiness were calculated as sum scores across relevant items; in addition, the total sum score was applied. Lower scores imply more symptoms of sleep problems.

As to other symptoms of acute Covid-19, we applied a previously developed inventory for adolescents with chronic fatigue syndrome consisting of 24 common symptoms graded on five-point Likert scales from “never/rarely present” to “present all of the time” [10-12]. These variables had a total range from one to five; higher scores imply more severe symptom burden.

The Hospital Anxiety and Depression Scale (HADS) is a validated questionnaire for charting symptoms of depression and anxiety [13, 14]. It consists of 14 items rated zero to three on Likert scales, allowing computation of sub-scores for depression and anxiety symptoms.

The Positive and Negative Affect Schedule (PANAS-SF) consists of five items addressing negative affects (ashamed, anxious, nervous, hostile, upset). The items are scored on 5-point Likert scales [15].

The Brief Illness Perception Questionnaire consists of eight items scored on 10-points Likert scale [16].

The Body Vigilance Scale (BVS) charts a total of 15 sensations in terms of how much attention is given to them [17].

The Pediatric Quality of Life Inventory (PedsQL) is translated and validated for the Norwegian population [18, 19]. It charts quality of life by 23 items scored on five-point Likert scales, each point valued zero, 25, 50, 75 or 100.

*Traits*

NEO-FFI-30 charts personality dimensions according to the ‘Big Five’ model of personality. In the present project, six items related to the neuroticisms axis are included; they are charted on 5-point Likert scales [20].

The Penn State Worry Questionnaire (PSWQ) is a validated questionnaire for measuring worrying tendencies [21]. It consists of 16 items rated on five-point Likert scales.

The Toronto Alexithymia Scale (TAS-20) subscale ‘Difficult identifying feelings’ is applied in the present project. A total of seven items is graded on 5-point Likert scales [22].

The UCLA loneliness scale consists 20 items scored on 4-point Likert scales; total sum scores ranges from 20 to 80 [23].

The General Self-Efficacy Scale consists of six items scored on 4-point Likert scales [24].

The Life Event Checklist (LEC) is a validated questionnaire for assessing both positive and negative life events [25]. The inventory lists 48 events; the respondents are asked to mark each of them as positive or negative, and grade their impact on four-point Likert scales. Also, the respondents were allowed to list additional events.

**Functional testing**

*Spirometry*

Spirometry was conducted to measure the forced vital capacity (FVC) and the forced expiratory volume in one second (FEV1) (EasyOne® Air spirometer; EasyOne Connect software, NDD Medizintechnik AG, Switzerland). The ratio of FEV1/FVC was calculated. Procedures were executed according to the American Thoracic Society (ATS) and European Respiratory Society (ERS) guidelines, and recordings that did not adhere to technical quality requirements were excluded from the main result analysis [26]. The Global Lung Function Initiative (GLI) network reference values were used to calculate the percentage of predicted values and the lower limit of normal (LLN) [27].

*Assessment of autonomic cardiovascular control*

A 5-minute ECG recording was performed applying The Bittium Faro 360® device (Bittium Corporation, Oulu, Finland). The participants were laying supine in calm surroundings, and with a pleasant ambient temperature. The recordings were subsequently subjected to heart rate variability analyses providing both time-domain and frequency-domain indices. In the frequency-domain, vagal (parasympathetic) activity is the main contributor to high-frequency (HF) variability of heart rate, whereas both vagal and sympathetic activity contributes to low-frequency (LF) variability [28]. The LF/HF ratio is considered an index of sympathovagal balance.

*Neurocognitive tests*

The digit span test from the Wechsler Intelligence Scale for Children, 4th edition (WISC-IV) [29] were used for assessment of verbal or auditory working memory [29]. The examiner reads aloud strings of random digits (approximately one digit per second). The test starts with two random numbers, increasing with one random number every other string. The digit span forward condition requires the test person to repeat the digits in the same order as heard; for digit span backward, the test person is required to repeat the digits in reverse order. Each answer is scored 1 (correct) or 0 (incorrect). When both strings in a pair (i.e. two strings of equal length) are answered incorrectly, the test is discontinued. Total scores are the sum of correct answers for both the forward and the backward condition.

The Hopkins Verbal Learning Test-Revised (HVLT-R) [30] is a test of verbal learning, delayed recall, and recognition [30]. The examiner reads aloud a list of 12 words, and the participant is asked to repeat as many words as possible in three consecutive trials; sum score of remembered words (0-36) in the three trials altogether is as measure of verbal learning. After 20 minutes, the participant is asked to recall the same 12 words; the number of remembered words (0-12) is a measure of delayed verbal memory. Finally, the examiner reads aloud 24 words, where 12 of these are identical to the previous list of words; the number of correctly recognized and falsely recognized words is recorded separately.

The Attention Bias test of automatic biases towards disease-associated words is specifically designed to address skewed attentions in chronic fatigue syndrome (CSF) patients [31]. It consists of a computer-based visual-probe task where disease-associated words are contrasted with neutral words.

The Function Acquisition Speed Test (FAST) is a computer-based test that similarly uses disease-related and neutral words to capture the strength of implicit associations between these words as determined by each individual’s previous learning history [32].

**Substudies**

*Cardiology substudy*

A total of 64 cases and controls were consecutively included in a cardiology sub-study at baseline featuring detailed echocardiographic examination. The aim of this substudy is to assess the impact on heart function from COVID-19 in the acute phase of the infection (ie .2-3 weeks after symptom onset) as well as on a long-term basis. Examinations was carried out by an experienced cardiologist using GE Vingmed E95 scanner (GE Vingmed Ultrasound, Horten, Norway) with adult 2D and 3D probes. In addition to the 64 participants included at baseline and followed prospectively, we will also perform echocardiography in a total of 90 individuals (cases and controls) at 6 months follow-up only, in order to increase statistical power for cross-sectional analyses.

*Brain fMRI substudy*

At 6 months follow-up, study participants are invited to a brain fMRI substudy. This substudy is integrated in the BRAINMINT project (cf. <https://www.sv.uio.no/psi/english/research/projects/brainmint/index.html>), and includes a resting state recording as well as a fatigue inducing task applying the multiple object tracking (MOT) paradigm. The total number of included participants in this substudy depends on MRI scanner availability, but we aim for at least 300.

*Qualitative sub-study*

At 6 months follow-up, a total of 25 individual who are 12-18 years of age and suffer from persistent symptoms after COVID-19 are invited to a qualitative sub-study addressing coping, coping beliefs and hope. The participants are recruited based on purposive sampling, and invited to a semi-structured interview lasting about one hour.

**Results – supplementary details**

| **Suppl. Tab. 1. Spirometry with less rigorous exclusion criteria** | | | | | | |
| --- | --- | --- | --- | --- | --- | --- |
|  | *COVID-19 (n=377)* |  | *Non-COVID (n=104)* |  | *p-value** |  |
| FVC, L - mean (SD) | 4.1 (0.99) |  | 4.2 (0.86) |  | 0.720 |  |
| Confidence interval | 4.0 to 4.2 |  | 4.0 to 4.3 |  |  |  |
| FVC, % of predicted - mean (SD) | 97.2 (12.4) |  | 98.8 (10.8) |  | 0.213 |  |
| Confidence interval | 96.0 to 98.5 |  | 96.8 to 100.9 |  |  |  |
| FVC < LLN - no. (%) | 27 (7.2) |  | 3 (2.8) |  | 0.099 |  |
| FEV1, L - mean (SD) | 3.5 (0.79) |  | 3.6 (0.71) |  | 0.633 |  |
| Confidence interval | 3.4 to 3.6 |  | 3.4 to 3.7 |  |  |  |
| FEV1, % of predicted - mean (SD) | 96.0 (12.7) |  | 97.4 (10.7) |  | 0.304 |  |
| Confidence interval | 94.7 to 97.3 |  | 95.3 to 99.4 |  |  |  |
| FEV1 < LLN - no. (%) | 33 (8.8) |  | 3 (2.8) |  | **0.038** |  |
| FEV1:FVC ratio - mean (SD) | 0.86 (0.074) |  | 0.86 (0.066) |  | 0.943 |  |
| Confidence interval | 0.85 to 0.87 |  | 0.85 to 0.87 |  |  |  |
| FEV1:FVC ratio < 0.7 - no. (%) | 12 (3.2) |  | 3 (2.8) |  | 1.000 |  |
| *Based upon Student t-tests, Chi-square test, and Fisher's excact test, as appropriate. SD=standard deviation; FVC=forced vital capacity; LLN=lower limit of normal; FEV1=forced expiratory volume 1 second. P-values ≤0.05 are indicated with **bold red**. | | | | | | |

**References**

1. *Standard Classification of Occupations. Oslo: Statistics Norway.* 1998.

2. Chalder, T., et al., *Development of a fatigue scale.* J Psychosom Res, 1993. **37**(2): p. 147-53.

3. Godfrey, E., et al., *Chronic fatigue syndrome in adolescents: do parental expectations of their child's intellectual ability match the child's ability?* J Psychosom Res, 2009. **67**(2): p. 165-8.

4. Tanaka, M., et al., *Reliability and validity of the Japanese version of the Chalder Fatigue Scale among youth in Japan.* Psychol Rep, 2008. **103**(3): p. 682-90.

5. Loge, J.H., O. Ekeberg, and S. Kaasa, *Fatigue in the general Norwegian population: normative data and associations.* J Psychosom Res, 1998. **45**(1): p. 53-65.

6. White, P.D., et al., *Comparison of adaptive pacing therapy, cognitive behaviour therapy, graded exercise therapy, and specialist medical care for chronic fatigue syndrome (PACE): a randomised trial.* Lancet, 2011. **377**(9768): p. 823-36.

7. Bedree, H., M. Sunnquist, and L.A. Jason, *The DePaul Symptom Questionnaire-2: A Validation Study.* Fatigue, 2019. **7**(3): p. 166-179.

8. Klepstad, P., et al., *The Norwegian brief pain inventory questionnaire: translation and validation in cancer pain patients.* J Pain Symptom Manage, 2002. **24**(5): p. 517-25.

9. Akerstedt, T., et al., *Disturbed sleep in shift workers, day workers, and insomniacs.* Chronobiol Int, 2008. **25**(2): p. 333-48.

10. Wyller, V.B., et al., *Sympathetic cardiovascular control during orthostatic stress and isometric exercise in adolescent chronic fatigue syndrome.* Eur J Appl Physiol, 2008. **102**(6): p. 623-32.

11. Sulheim, D., et al., *Disease mechanisms and clonidine treatment in adolescent chronic fatigue syndrome: a combined cross-sectional and randomized clinical trial.* JAMA Pediatr, 2014. **168**(4): p. 351-60.

12. Wagner, D., et al., *Psychometric properties of the CDC Symptom Inventory for assessment of chronic fatigue syndrome.* Popul Health Metr, 2005. **3**: p. 8.

13. Zigmond, A.S. and R.P. Snaith, *The hospital anxiety and depression scale.* Acta Psychiatr Scand, 1983. **67**(6): p. 361-70.

14. Bjelland, I., et al., *The validity of the Hospital Anxiety and Depression Scale. An updated literature review.* J Psychosom Res, 2002. **52**(2): p. 69-77.

15. Thompson, E., *Development and validation of an internationally reliable short-form of the positive and negative affect schedule (PANAS).* Journal of cross-cultural psychology, 2007. **38**: p. 227-242.

16. Broadbent, E., et al., *The brief illness perception questionnaire.* J Psychosom Res, 2006. **60**(6): p. 631-7.

17. Schmidt, N.B., D.R. Lerew, and J.H. Trakowski, *Body vigilance in panic disorder: evaluating attention to bodily perturbations.* J Consult Clin Psychol, 1997. **65**(2): p. 214-20.

18. Varni, J.W., et al., *The PedsQL as a patient-reported outcome in children and adolescents with fibromyalgia: an analysis of OMERACT domains.* Health Qual Life Outcomes, 2007. **5**: p. 9.

19. Reinfjell, T., et al., *Measuring health-related quality of life in young adolescents: reliability and validity in the Norwegian version of the Pediatric Quality of Life Inventory 4.0 (PedsQL) generic core scales.* Health Qual Life Outcomes, 2006. **4**: p. 61.

20. Korner, A., et al., *[Personality assessment with the NEO-Five-Factor Inventory: the 30-Item-Short-Version (NEO-FFI-30)].* Psychother Psychosom Med Psychol, 2008. **58**(6): p. 238-45.

21. Meyer, T.J., et al., *Development and validation of the Penn State Worry Questionnaire.* Behav Res Ther, 1990. **28**(6): p. 487-95.

22. Bagby, R.M., G.J. Taylor, and J.D. Parker, *The Twenty-item Toronto Alexithymia Scale--II. Convergent, discriminant, and concurrent validity.* J Psychosom Res, 1994. **38**(1): p. 33-40.

23. Russell, D., L.A. Peplau, and C.E. Cutrona, *The revised UCLA Loneliness Scale: concurrent and discriminant validity evidence.* J Pers Soc Psychol, 1980. **39**(3): p. 472-80.

24. Romppel, M., et al., *A short form of the General Self-Efficacy Scale (GSE-6): Development, psychometric properties and validity in an intercultural non-clinical sample and a sample of patients at risk for heart failure.* Psychosoc Med, 2013. **10**: p. Doc01.

25. Johnson, J. and S. McCutcheon, *Assessing life stress in older children and adolescents: Preliminary findings with the life event checklist. In I.G. Sarason & C.C. Spielberger (Eds),.* Stress and anxiety, 1980. **7**: p. 15.

26. Graham, B.L., et al., *Standardization of Spirometry 2019 Update. An Official American Thoracic Society and European Respiratory Society Technical Statement.* Am J Respir Crit Care Med, 2019. **200**(8): p. e70-e88.

27. Quanjer, P.H., et al., *Multi-ethnic reference values for spirometry for the 3-95-yr age range: the global lung function 2012 equations.* Eur Respir J, 2012. **40**(6): p. 1324-43.

28. *Heart rate variability. Standards of measurement, physiological interpretation, and clinical use. Task Force of the European Society of Cardiology and the North American Society of Pacing and Electrophysiology.* Eur Heart J, 1996. **17**(3): p. 354-81.

29. Wechsler, D., *Wechsler Intelligence Scale for Children 4^th^ ed. San Antonio (WISC-IV): Psychological Corporation.* 2003.

30. Benedict, R., et al., *Hopkins Verbal Learning Test-Revised: Normative data and analysis of inter-form and test-retest reliability.* Clin Neuropsychol, 1998. **12**: p. 13.

31. Hughes, A.M., et al., *An attention and interpretation bias for illness-specific information in chronic fatigue syndrome.* Psychol Med, 2017. **47**(5): p. 853-865.

32. Cummins, J. and B. Roche, *Measuring differential nodal distance using the function acquisition speed test.* Behav Processes, 2020. **178**: p. 104179.
